# Provision of mental health and psychosocial support services to health workers and community members in conflict-affected Northwest Syria: a mixed-methods study

**DOI:** 10.1186/s13031-023-00547-4

**Published:** 2023-10-04

**Authors:** Ibrahim R. Bou-Orm, Marianne Moussallem, Joelle Karam, Manuel deLara, Vinod Varma, Karin Diaconu, Murat Can Birand Apaydin, Rafael Van den Bergh, Alastair Ager, Sophie Witter

**Affiliations:** 1 ReBUILD for Resilience, Institute for Global Health and Development, Queen Margaret University, Edinburgh, UK; 2 Higher Institute of Public Health, Faculty of Medicine, Saint Joseph University of Beirut, Beirut, Lebanon; 3 World Health Organization, Gaziantep, Turkey; 4 Attacks On Health Care Initiative, World Health Organization, Geneva, Switzerland; 5 Institute for Global Health and Development, Queen Margaret University, Edinburgh, EH21 6UU UK

**Keywords:** Service delivery, Health system resilience, Humanitarian settings, Conflict and health, Mental health

## Abstract

**Background:**

Northwest Syria (NWS) is a conflict area with challenging political, economic, demographic and social dynamics. The region has a high number of internally displaced persons with increasingly disrupted delivery of basic services, including healthcare. Mental health needs have been increasing in the region while the infrastructure and capacity of the health sector has been negatively affected by the conflict. This study aimed to explore the provision of mental health and psychosocial support (MHPSS) services to communities in NWS (including healthcare workers) and to assess the experiences of beneficiaries with MHPSS services.

**Methods:**

The study followed a mixed-methods research design that included qualitative and participatory methods (44 semi-structured interviews and a group model building workshop with 15 participants) as well as a survey with 462 beneficiaries.

**Results:**

Findings suggested an improvement of MHPSS services in the region over the last few years due to the creation of a specific Technical Working Group for MHPSS that contributed to assessment of community needs and support of the MHPSS response. The key elements of this response were: (1) training non-specialized health workers to address the shortage in specialized providers; (2) securing funding and coordination of services between different organizations; and (3) addressing gaps in the availability and geographical distribution of other needed resources, such as medicines. While those elements contributed to improving access to services and the quality of services—especially among health workers seeking MHPSS services—findings suggested gaps in the sustainability of services and a need to scale up those interventions in an integrated approach.

**Conclusion:**

The study findings add to the evidence base on the challenges in scaling up MHPSS interventions and their long-term sustainability concerns. Priority actions should address the intermittent funding of the MHPSS response, incorporate MHPSS outputs and outcomes in the reimbursement of routine services, improve coordination between health partners and non-health actors in order to expand the scope of MHPSS response, and address the inequitable availability of resources in the region.

## Background

The burden of mental health problems has grown considerably over the last decade. The latest Global Burden of Diseases estimates rank this category of health problems at the 7th place in terms of disability-adjusted life years, noting a consistent increase in prevalence over time [1]. Despite this high burden, the World Health Organization (WHO) estimates a considerable treatment gap, especially in low- and middle-income settings: more than 76% of people living with mental health problems in those settings do not receive treatment [2]. Conflict further exacerbates this gap, both by increasing the burden of disease and by compromising the modalities to provide appropriate care.

The most updated WHO estimates reveal that the prevalence of mental health problems (including depression, anxiety, and posttraumatic stress disorders (PTSD)) among conflict-affected populations is around 22% at any point of time [3]. However, this is likely an underestimate of actual burden, given severe stigmatization of such conditions. Moreover, care provision is challenging in such settings because of insecurity and violence, which impacts on accessibility of health services and on the infrastructure and other resources of the health systems (e.g., human resources) needed to maintain their ability to deliver services [4]. Attacks on healthcare workers, as well as their high exposure to traumatic events during conflict, also lead to increased health needs (including mental health needs) among healthcare workers themselves and compromise the health system as a whole [5–7].

Since March 2011, Syria has been facing the largest refugee and displacement crisis in the world, with more than 10 million displaced persons (5.6 million Syrians have fled the country and 6.6 million have been internally displaced), and around 400,000 deceased and 11.1 million in need of humanitarian assistance to date [8, 9]. Northwest Syria (NWS), which is under control of armed opposition groups, hosts the majority of internally displaced people (IDPs) and receives humanitarian support by cross-border aid activities from Turkey [10]. Since the beginning of the crisis, the overall health response in NWS has been coordinated by WHO, given the challenging governance of the health system in the area.

In February 2016, an MHPSS Technical Working Group (MHPSS TWG) was created and became the coordination body between different actors steering the overall direction of MHPSS interventions in NWS. The MHPSS TWG includes psychiatrists, psychologists, and representatives of the organizations active in the field of MHPSS. It provides technical guidance and capacity building for actors. A range of innovative interventions have been implemented since 2016 to scale up the provision of MHPSS in the region to address community needs [11, 12]. These interventions followed the Mental Health Gap Action Programme (mhGAP) strategy [13] and aimed to overcome gaps in specialists by building the capacity of general practitioners and non-skilled workers to conduct non-specialized mental health diagnosis, management and treatment, as well as training Psychosocial Workers (PSWs) to provide non-specialized psychosocial support, including a focus on self-care and staff care. While these interventions were aimed at providing access to MHPSS services to the general population, anecdotal reports suggest that healthcare workers in particular may have benefited from such services.

This study aimed to understand the implementation process of the MHPSS response and to identify potential gaps and barriers in its implementation. It also explored the responsiveness of MHPSS services towards host communities, internally displaced persons, and healthcare workers, with a special focus on access to MHPSS care, acceptability of MHPSS initiatives, and user satisfaction.

## Methods

### Study design and setting

This study followed a mixed-methods approach that included document review, qualitative and participatory methods (such as semi-structured interviews and group model building workshops) as well as quantitative methods (surveys). This study was conducted in three regions located in two different governorates in NWS: Afrin and Azaz located in Aleppo governorate and Idlib, the capital of Idlib governorate.

### Semi-structured interviews

#### Characteristics of participants

We conducted a total of 44 interviews with (1) representatives of non-governmental organizations (NGOs) supporting MHPSS initiatives and members of the MHPSS TWG; (2) health professionals (doctors, midwives, and other PSWs) working in the different facilities providing specialized and non-specialized MHPSS services; and (3) beneficiaries from host communities and IDPs (including health workers affected by attacks). The characteristics of the key informants are presented in appendix 1.

We followed a convenience and purposive sampling approach for interviews, with the inclusion criteria for health workers and MHPSS staff being: (1) aged above 18 years, (2) being in post for at least one year. As for beneficiaries including health care workers seeking healthcare support, participants were identified by a MHPSS provider and recruited based on the following criteria: (1) being adult; (2) living with mental health problems; (3) receiving treatment for at a least 3 months in the facility; (4) ability to consent to participate in the study. We excluded those who might be negatively affected by the participation (e.g. people with uncontrolled mental health problems), based on the judgment of the MHPSS expert. Recruitment of participants and interviews were conducted by trained data collectors until data saturation was reached. For beneficiaries, providers first asked potential participants if they are interested to participate in a research study on MHPSS service delivery and introduced them to the research team, who provided information about the study and took consent.

#### Description of data collection tool and process

The interviews were conducted by trained data collectors, in Arabic or English, face-to-face or remotely (depending on the preferences of the interviewees), audio-recorded (if consent obtained) and transcribed directly in English. The interviews lasted between 30 to 45 min. Oral consent was obtained and recorded for all interviews at the beginning of the interview as ‘consent secured’.

As for the data collection tool, the research team developed three different topic guides targeting the different categories of participants and based on different frameworks. We used the Inter-Agency Standing Committee (IASC) intervention pyramid [14] and the WHO ExpandNet framework to assess the scaling up of interventions. The latter reflects on the interaction between elements such as the innovation, the user organization, the environment, the resource team, and the scaling up strategy and details the needed attributes of each element [15]. We drew on WHO’s frameworks for health system building blocks [16], on integrated people-centered health services [17] and WHO’s Quality of Care framework for fragile and conflict affected settings [18] to explore aspects of service delivery and to investigate how the current health system in North-West Syria has supported and benefited from the implementation of MHPSS service delivery.

The topic guides included questions grouped under five main categories:

1. Rationale and process of MHPSS interventions and service delivery
2. Importance of service integration and status of available resources
3. Access to MHPSS services
4. Quality of care
5. Acceptability of non-specialist services

#### Analysis

The interviews were transcribed directly in English, and transferred to the researchers for data analysis. Researchers conducted a thematic analysis using both inductive and deductive approaches. They created initial codes to code all interview data. Afterwards, the initial codes were then combined and categorized to extract the main themes.

### Group model building (GMB) workshop

Group model building (GMB) is a systems thinking method employed in health systems research to enhance collective understanding and decision-making processes. It involves bringing together diverse stakeholders, such as policymakers, practitioners, and community members, to collaboratively construct dynamic models that represent the intricacies of complex issues within health systems. Through facilitated discussions and interactions, group model building enables participants to collectively identify system components, relationships, and feedback loops, fostering a holistic understanding of the system's behavior. This participatory approach facilitates knowledge sharing, mutual learning, and the exploration of potential policy interventions, leading to more effective strategies for addressing complex health challenges.

#### Characteristics of participants

A total of 15 participants, including health managers and service providers (doctors, nurses, and psychosocial workers (PSWs)) participated in the workshop. Inclusion criteria were: (1) being in post for at least a year; (2) working in the regions of interest. Participants were recruited using convenience and purposive sampling. The characteristics of the participants are presented in Appendix 1.

#### Description of material and data collection process

We conducted one group model building (GMB) workshop online based on a series of scripts outlining the workshop activities; and based on the theoretical frameworks guiding this research. The main variables that the team sought to map out through the workshop were: mental health needs, community health-seeking behaviors, service provision, care quality, referrals, human resource availability and skills, different aspects of coordination including intra and inter-facilities coordination, and multi-level coordination involving all the MHPSS interventions bodies such as TWG, different NGOs and stakeholders active on the ground.

The research team presented the aim of the research and requested verbal consent from the participants to record the sessions at the beginning of the workshop. The workshop was conducted in Arabic on MS Teams due to the coronavirus disease (COVID-19) pandemic. The total duration of the workshop was about 6 h over 2 half-days.

#### Analysis

The workshop generated diverse material: drawings, audio recording and causal loop diagrams (CLD) summarizing the workshop discussions. The analysis of the GMB data was iterative: researchers took notes during the workshop and transcribed the discussions using the recordings of the sessions. Based on these inputs, the team reviewed and cleaned the developed model which was then shared with the participants together with a summary of the discussions. The final version is presented in the summary of findings in the discussion section of this paper.

## Survey

### Characteristics of participants

The target population was MHPSS service users, including healthcare providers and other beneficiaries receiving services in the WHO-supported facilities. We selected facilities based on the recommendations of the WHO team to ensure diversity in terms of geographical location. The sample size was calculated using a stratified sampling approach with a probability of 50% to secure the maximum variability in responses, a margin of error of 0.05 and a design effect of 1.2. A total of 458 participants were recruited based on the same inclusion and exclusion criteria as for the interviews with beneficiaries.

### Description of data collection tool and process

We prepared the survey questionnaire in English, and then translated it into Arabic before pilot testing with 10 individuals from the target group. This allowed linguistic validation by investigating the equivalence of concepts between the two versions of the questionnaire. It also helped ensure cultural validation such as the appropriateness of wording, and potential misinterpretation. The final questionnaire consisted of seven main sections:

1. Socio-demographic characteristics of participants
2. Demand and access to primary mental healthcare services
3. Perceived quality of care
4. Acceptability of MHPSS and the task-shifting approach
5. Patient satisfaction
6. Mental health outputs
7. Beneficiaries’ needs.

As for the recruitment of participants, an MHPSS specialist at the sampled health facilities located in Azaz, Afrin and Idlib identified beneficiaries who were eligible to participate in the study and checked their willingness to be approached by data collectors. The data collectors asked the participants for their oral consent to participate in the study and offered technical support to participants to fill in the survey. The surveys lasted approximately 30 min. Data collection occurred in the health facilities so any potential distress could be handled by health professionals.

### Statistical analysis

First, we conducted a descriptive analysis: categorical variables were presented as frequencies and percentages, and numeric variables as means and standard deviations. Bivariate analyses were also performed using chi-square tests and t-tests. A *p*-value ≤ 0.05 was considered statistically significant.

### Ethical considerations

The full study protocol, including all data collection activities (GMB workshops, semi-structured interviews and the survey), was reviewed and cleared by the ethics committees of the St. Joseph University of Beirut, Lebanon (reference number USJ-2021–222) and Queen Margaret University Edinburgh, UK. Data collectors were trained in basic interviewing skills as well as ethical principles, including privacy, confidentiality, informed and voluntary participation and the best interest of interviewees. Moreover, they were all introduced to care referral pathways and the local context, including gender and cultural sensitivity. In all data collection phases, participants had full autonomy to stop their participation at any time without providing any explanation. All datasets were initially stored on encrypted password-protected laptops and then transferred to university servers.

## Results

### Appraising MHPSS service provision: findings from qualitative interviews and the group model building workshop

#### Increasing needs and neglected MHPSS provision

Participants in the GMB workshop and interviews highlighted that MHPSS service provision in NWS was neglected, unorganized and very limited until the creation of a specific technical working group for MHPSS in February 2016 and the subsequent activities in 2017–2018 to evaluate the burden of mental ill health and the needed response.

“A needs assessment was carried out at the end of 2015. Prior to that, there were no MHPSS interventions or facilities. If there were any mental health services offered, they were individual initiatives.” – Mental Health Coordinator

Before 2016, the focus of health services was on physical health rather than mental health, despite the high level of violence and other stressors, according to GMB participants. In terms of available services, they described a localized and limited availability of services at the borders with Turkey, where active organizations were operating. The situation inside NWS was fragile with insufficient resources in terms of specialized MHPSS providers (as a result of emigration of skilled specialists in 2016–2017) and medicines. Interviewed health providers also reported that communities rarely knew about MHPSS services and demands for these services were low but increased over the years. Many health providers noted that beneficiaries considered in recent years their mental health as a top priority, at the same level as other basic needs such as shelter and security.

“MHPSS services are considered to be good more or less; although, they are insufficient and do not cover all the needs.” – Mental Health Coordinator

#### MHPSS response in NWS: perceptions of TWG members, NGO representatives and health providers

Participants in the workshop and interviews noted that the main element of the MHPSS response was the delivery of training (especially in 2018) to mitigate the shortage in specialized MHPSS workforce and stressed the relevance of these interventions. Interviewed NGO representatives explained that those trainings initially targeted general practitioners, using the mhGAP training approach with close supervision. They also noted that PSWs received training on identification and referral skills, using manuals developed by the TWG. Some participants mentioned that Community Workers (CWs) have also been trained on Psychological First Aid (PFA) delivery and referral procedures and this training contributed to improving the accessibility and acceptability of services by beneficiaries as CWs have a close understanding of the community they serve and are trusted by other community members.

Health providers who participated in the GMB workshop considered that the scope of trainings was not comprehensive enough to allow doctors to treat the whole range of mental health problems. They reported a disease-focused training approach which was sometimes repeated several times. Interviewed PSWs did not show same levels of confidence regarding their skills and their ability to diagnose and provide MHPSS counselling and treatment.

“mhGAP training was necessary to address the shortage of specialized mental health professionals and the growing number of needs.” – Mental Health Coordinator

In addition to training, participants acknowledged the role of service mapping as a facilitator of referral operations as it displayed the distribution of facilities operating in the region. Those referrals usually included beneficiaries who needed services beyond the scope of the providers’ interventions. Participants identified the referral process as very crucial as non-specialists can usually identify cases and provide basic interventions but need to refer many cases to other facilities. However, interviewed providers noted that other non-MHPSS providers do not know about the referral process and available facilities for referral.

NGO representatives and TWG members noted the implementation of other interventions including the Problem Management Plus (PM+) programme and training on post-natal depression and suicide prevention. They also noted a change in service delivery during the COVID-19 pandemic through the introduction of online sessions. They also identified enablers to improve the accessibility and acceptability of MHPSS services. Several participants in this category mentioned awareness activities to improve the knowledge of communities about mental health problems and the availability and benefits of MHPSS services, as well as the assistance of community leaders (e.g., school directors and religious leaders), radio stations and social media platforms to disseminate those materials and spread medical and service-related information among community members. They identified the use of Mobile Medical Units (MMUs) to improve access to services among those who have geographical barriers to care (e.g., long distance to facilities requiring high transportation expenses). Some participants considered the MMU alternative as an opportunity to access those affected by financial and security constraints. However, one NGO representative participant raised concerns about the lack of confidentiality and privacy in MMUs or other used locations by the MMU team (e.g. tents).

“We conduct awareness campaigns in schools and explain about the benefits of MHPSS services, when and how to access them. Some students might express their worries, and this is when we refer them to the services needed.” Mental Health Coordinator

#### Coverage and relevance of MHPSS interventions and services

Health providers and NGO representatives reported an improvement in the availability of services due to efforts to reduce the gap in human resources. However, they noted that the current coverage of services and available resources (mainly human resources) are still insufficient to meet all the needs of communities. For instance, they noted challenges to collaborate with other sectors to address the stressors among affected community members and to provide advanced mental health care. Participants in the GMB workshop stressed the lack of psychology-related services (e.g. cognitive behavioral therapies) due to limited numbers of psychology graduates and trainings. A few interviewed participants from NGOs and the TWG reported that MHPSS training targeted personal from a wide range of backgrounds (e.g., education, social sciences) due to the urgency to increase human resources in the region.

Other interviewed participants identified relevant interventions such as peer-to-peer support which were created to help providers in need of mental health assistance because of their workload and environment. Healthcare providers also benefit like other beneficiaries from MHPSS services, including awareness sessions within their facilities (posters, distributed posters and flyers).

“As for the needs of the staffs, especially after the emergency, I do not have any knowledge of the different centres, but even for us, as specialists in providing PSS services, we have high pressure in normal conditions and are charged with a lot of work. The organization does not provide facilities to relieve stress such as holidays and self-care, so that I was suffering from great professional pressure in the past period and I was about to leave the job, however we are provided with supportive information and some updates by a direct supervisor.” – PSW

In terms of adequacy to cover different community groups, NGO representatives and TWG members noted that MHPSS services are intended to be for everyone (including healthcare workers (HCWs)), and providers make every effort to provide assistance to all groups. Interviewed participants also noted the absence of any discrimination in providing services to both women and men. However, they highlighted community-related factors that may affect the ability of women to access MHPSS services. Examples included fear of the reaction of male relatives (e.g., husbands) among women or transportation challenges.

Different accounts were reported regarding the availability of services for other vulnerable groups. For instance, many participants highlighted the gap in specialized services for children and people with disabilities.

Participants also reported an unequitable geographical distribution of facilities and MHPSS services in the region, which reduces access to care. One participant linked this situation to the preference of donors to support services in stable locations to ensure sustainability. Participants in the GMB workshop reported a decline in MHPSS services during the COVID-19 pandemic because of the closure of several specialized facilities.

“In the northern regions of Aleppo and eastern Idlib, there is a good availability of MHPSS services, while in the western regions of Idlib and other areas, services and providers are limited, due to the military operations that may take place. Even donors do not provide support in these regions, as the sustainability of services is questionable.” – Mental Health Coordinator

#### Sustainability of services and continuity of care

Participants described sustainability of interventions in NWS as uncertain. Interviewed NGO representatives and TWG members noted an intermittent delivery of services, which is caused by the short-term type of funding (common duration of 6–12 months) with no guarantee of renewal. The ultimate result of financial instability would be the interruption of services for beneficiaries, despite the efforts to refer them to other facilities. Interviewed health providers confirmed this observation and also reported the absence of strategies to mainstream MHPSS interventions between different partners. They recommended more coordination to improve the compatibility and complementarity of interventions.

“…the psychiatric clinic where I work will stop in a few days and the continuity of services is still not guaranteed.” mhGAP physician

Participants noted a limited integration of MHPSS services because of the lack of earmarked funding for MHPSS. Interviewed participants highlighted that mhGAP-trained doctors are usually overwhelmed with addressing the physical health needs of communities and cannot ensure an adequate pathway of care for mental health needs. An interviewed NGO representative explained this practice as being due to (1) the lack of financial incentives for non-specialized providers, affecting their performance in case identification, management and referral; (2) the limited confidence of providers in services delivered in other facilities. Participants in the GMB workshop confirmed this, highlighting their reluctance to refer potential cases to centers due to lack of confidence in the expertise of providers there. Other participants noted that providers are not always committed to the referral process, to ensure a retention of beneficiaries in their system. As for the referral process itself, different accounts emerged: several informants mentioned service mapping as a useful tool for assisting with referral procedures, while others expressed dissatisfaction with the lack of a standardized form for referrals.

#### Importance of service integration and status of available resources

Our participants stated that the integration of MHPSS services within sustainable care models would improve the MHPSS response towards a more comprehensive and continuous approach, as well as access of communities to those services. For instance, participants highlighted that the integration of MHPSS services in PHC settings would help communities to overcome stigma-related barriers as beneficiaries are identified by their social network as users of primary healthcare (PHC) services in general, instead of mental health services.

However, interviewed participants identified limited arrangements in terms of resources and management for the integration and scale up of services. Interviewed health providers saw the current infrastructure of health facilities as an obstacle due to the lack of space to provide MHPSS services and protect privacy and confidentiality. Moreover, participants re-emphasized limited MHPSS-related human resources as well as the need to incentivize mhGAP trained doctors to deliver MHPSS consultations as part of their routine work. When asked about the current capacity of specialized human resources, participants noted that only three psychiatrists and a limited number of psychologists are available in NWS. Participants considered the task-shifting approach an effective strategy to mitigate the shortage of human resources, but some raised a concern about the quality of services delivered by lower-skilled providers.

When prompted to reflect on the medication supply and availability, interviewed participants valued the role of WHO in securing the availability MHPSS drugs and putting in place a dispensing mechanism of essential medicines according to mhGAP protocols and to the needs of facilities every three months. However, health providers noted prolonged shortages of MHPSS drugs which was affecting clinical outcomes and the experiences of beneficiaries who had to rely on private pharmacies (including those in Turkey) to get medicines in the WHO-list of medicines. This observation was at odds with the account of some interviewed NGO representatives and TWG members, who noted that the stock is satisfactory, and the problem is with the limited availability of eligible providers to prescribe medicines.

Participants also highlighted challenges in finance and governance. Participants reported that funding of MHPSS services is not prioritized by donors and is based on criteria that cannot always be met by organizations such as efficiency in service delivery and strength of their profiles in terms of completion of previous similar projects.

In terms of governance arrangements, many participants considered that the MHPSS–TWG helped in the standardization of practices and quality requirements, and mobilization of resources. However, there was no consensus about participation within this platform. Many interviewed TWG members considered that funding and technical activities were based on project proposals grounded in needs assessments and the identification of gaps in previously implemented interventions. However, health providers in the GMB workshop and the interviews reported a limited contribution of providers to the decision-making process and considered that the implementation of MHPSS interventions was donor-driven, rather than being the result of a participatory approach between actors or based on actual needs. Overall, this suggests the need to improve communication between decision-makers and providers in order to move towards a bottom-up planning process. In addition, interviewed health providers identified the need for more stewardship over the MHPSS response, given the multitude of health authorities in the region.

Participants highlighted the role of community engagement throughout intervention planning and execution as a determinant of sustainability. In addition to the contribution of communities to service delivery (as trained CWs) by delivering PFA, for instance, participants acknowledged the role of community leaders (e.g. religious leaders, camp leaders, school directors, local authorities’ representatives) to access communities and increase acceptance of mental health services.

Finally, participants reported that Monitoring and Evaluation (M&E) is important to check the adequacy of services and identify gaps in service delivery. They noted previous positive examples such as the supervision of non-specialized providers for 6 months while delivering MHPSS services and the availability of supervisors to intervene in case of advanced services, as well as M&E activities conducted by organizations and third-party organizations. Nonetheless, they described the evaluation process as insufficient and raised two challenges: remote follow-up by organizations from Turkey; and secondly, the absence of prioritization and adequate allocation of funding of M&E activities.

### Experiences of beneficiaries with MHPSS services in NWS: findings from the interviews

#### Access to MHPSS services

Beneficiaries reported easy access to MHPSS services in the facilities from which they were recruited. Nonetheless, they noted many barriers to MHPSS services in their communities, including stigma of mental health problems, the need to travel for long distances to get services, non-assistance to people with disabilities, and prejudice against mental health services (e.g., fear of being prescribed lifelong medicines that have a significant impact on their lives, or being diagnosed with mental health problems requiring hospitalization, etc.). Interviewed beneficiaries reported that the knowledge of MHPSS services—which can be acquired during the visits of community members to health facilities—and the positive experiences of other persons within their social networks (e.g. friends and neighbours) improved the health-seeking behaviour of people in need of MHPSS services.

“I learned about the programme through a neighbour who told me about how he had benefited from it, and it was convenient to access due to its proximity to my home.” – IDP

“I was at the health centre for a gynecologist appointment, and the PSW was holding a suicide awareness campaign, which piqued my interest. However, the problem is that the centre is far away, and my parents refused to let me seek the service.” – IDP

“I was concerned that because I am a member of the centre’s staff, news of my visit to a PSS centre would spread among my colleagues”- IDP

Interviewed health providers and NGO representatives confirmed these observations, but they reported a decrease in MHPSS stigma compared to previous years, due to increased community awareness about mental health problems and most importantly the benefits of seeking formal medical support. This category of participants reflected on the gender-related differences in access to care in different regions. The ability of women or men to seek services depends on the cultural norms and beliefs in their regions. A beneficiary reported that, in some areas, men would consider mental health problems as a weakness and refuse to seek help. A few healthcare workers who benefited from MHPSS services also reported the same barrier among their colleagues as well.

“I know about the services because I work at the same centre. When I arrived, they greeted me warmly, and it was because of this that I dared to ask for service. When I arrived, they did not make me feel weird” – Local resident/ paramedic

#### Quality of care

Beneficiaries reported that received services contributed to a significant improvement of their health status (including physical health), as well as their functionality and social life.

They also said that their providers have the necessary skills and are competent to deliver the services. They described them as good listeners, supportive, and respectful. Beneficiaries also noted that the providers explain their mental health problems and contributing factors and stressors, discuss the care plan, including desired and expected outcomes, and provide a follow-up plan.

“She outlined to me several treatment options and we decided on the one that was best for my situation. She told me as well about the benefits of these strategies” – Local resident

“She stressed the significance of following up on sessions, developing a strategy, and partnering on its implementation” – IDP

Beneficiaries commented that providers adapt the treatment strategy according to individuals’ experiences and inform them about positive and negative coping mechanisms to deal with their mental health problems.

This observation was confirmed by interviewed health providers and NGO representatives. Interviewed health providers noted that they regularly assess beneficiaries’ needs to track potential changes and to adjust treatments accordingly, based on discussions with beneficiaries. NGO representatives and TWG members stated that trainings focused on the development of providers’ communication skills and their ability to interact with beneficiaries to identify and address their needs and involve them in decision-making. However, they acknowledged that facilities sometimes fail to meet the expectations of some beneficiaries, who anticipate a quick and complete solution to their situations.

In addition to those features of service quality, beneficiaries and providers reflected on other aspects that relate to person-centredness. Most health providers noted that MHPSS services are tailored to meet the preferences of all beneficiaries regardless of their gender, socio-economic status, and residency status, which was confirmed by interviewed beneficiaries. For instance, facilities try to mitigate cultural barriers affecting women’s access to MHPSS by hiring both female and male healthcare workers. However, many participants reported that there are gaps in services for specific age groups or vulnerable groups (such as children). When prompted to discuss whether services are tailored according to the needs of health providers themselves, health providers reported that no specific measures (beyond the available services) are taken by facilities to mitigate the difficult work and context conditions of healthcare workers and to reduce stressors that can lead to mental health problems.

#### Acceptability of non-specialist services

Most beneficiaries agreed that they accept being followed up by non-specialist providers if they receive the needed services. In addition, most beneficiaries do not recognize the difference between specialized and non-specialized practitioners, according to interviewees from all categories (including beneficiaries). Moreover, NGO representatives reported that MHPSS providers like PSWs have the qualifications allowing them to deliver services (e.g. trainings, supervision). In our study, beneficiaries had a positive experience and reported a safe service by MHPSS providers regardless of their status.

“In my opinion anyone with a university degree and training can do the work. There is no obligation to be a specialist.” – IDP

Beyond acceptance of MHPSS services, which was considered generally good, beneficiaries reported a good satisfaction with MHPSS services. NGO representatives, TWG members and health providers noted that general adherence to treatment and follow up plans is an indicator of beneficiaries’ satisfaction.

### Beneficiary survey findings

A total of 462 individuals completed the survey, 40.9% of which were from Idlib, 31.0% from Azaz and 28.1% from Afrin. The sample included both residents (45.9%) and IDPs (54.1%), as well as healthcare workers who used MHPSS services (50.4%) and other beneficiaries (49.6%). The majority of participants were recruited from a general clinic in a PHC centre (37%), a mental health clinic in a hospital (31%) or a mental health clinic in a PHC centre (13%). Mean age was 33 years (SD = 7.9) and just over half of the sample were women. Most participants had a low socio-economic status. The socio-demographic characteristics of participants are detailed in Table 1 below.

**Table 1:** Socio-demographic characteristics of the sample of MHPSS beneficiaries in NWS (n = 462)

Variables	Healthcare workers (n, %)	Other beneficiaries (n, %)	Total (n, %)	*P*-value
Region				0.964
Afrin	67 (28.8%)	63 (27.5%)	130 (28.1%)	
Azaz	71 (30.5%)	72 (31.4%)	143 (31,0%)	
Idlib	95 (40.8%)	94 (41.0%)	189 (40.9)	
Facility from which the participants were recruited				**< 0.001**
Clinic in PHC centre	83 (35.6%)	89 (38.9%)	172 (37.7%)	
MH Clinic in PHC	39 (16.8%)	21 (9.1%)	60 (13.2%)	
MH Clinic in specialized centre	2 (0.9%)	0 (0%)	2 (0.4%)	
MMU	14 (6.0%)	25 (10.9%)	39 (8.5%)	
MH Clinic in Hospital	81 (34.8%)	62 (27.1%)	143 (31.4%)	
MH Clinic in specialized hospital	2 (0.9%)	32 (14.0%)	34 (7.5%)	
Residency status				0.264
Resident	113 (48.5%)	99 (43.2%)	212 (45.9%)	
IDP	120 (51.5%)	130 (56.8%)	250 (54.1%)	
Age categories				**< 0.001**
18–30	82 (36.9%)	94 (43.7%)	176 (40.3%)	
31–40	113 (50.9%)	71 (33.0%)	184 (42.1%)	
41–60	27 (12.2%)	47 (21.9%)	74 (16.9%)	
61–70	0 (0%)	3 (1.4%)	3 (0.7%)	
Age in years (mean ± SD; Range)	32.9 ± 6.3	33.2 ± 9.2	33 ± 7.9; 18–70	0.785
Gender				**0.004**
Female	125 (53.6%)	151 (65.9%)	276 (59.7%)	
Male	104 (44.6%)	78 (34.1%)	182 (39.4%)	
Prefer not to say	4 (1.7%)	0 (0%)	4 (0.9%)	
Marital status				0.239
Married	190 (81.5%)	168 (73.7%)	358 (78.7%)	
Widowed	3 (3.9%)	14 (6.1%)	17 (3.7%)	
Single	25 (10.7%)	33 (14.5%)	58 (12.7%)	
Separated/divorced	9 (3.9%)	13 (5.7%)	22 (4.8%)	
Educational level				** < 0.001**
Primary school level or less	8 (3.4%)	111 (48.5%)	119 (25.8%)	
Secondary School level	5 (2.1%)	66 (28.8%)	71 (15.4%)	
High school	20 (8.6%)	33 (14.4%)	53 (11.5%)	
University	200 (85.8%)	19 (8.3%)	219 (47.4%)	
Employment status				** < 0.001**
Employed	229 (98.7%)	31 (13.5%)	260 (56.3%)	
Unemployed	3 (1.3%)	197 (86%)	200 (43.3%)	
Retired	0 (0%)	1 (0.4%)	1 (0.2%)	
Crowding index (mean ± SD)	2.3 ± 1.1	3.2 ± 1.9	2.7 ± 1.7	** < 0.001**
Socio-economic status (SES)				**0.001**
Low SES	174 (74.7%)	194 (87.8%)	368 (81.1%)	
Middle SES	56 (24.0%)	26 (11.8%)	82 (18.1%)	
High SES	3 (1.3%)	1 (0.5%)	4 (0.9%)	

#### Access to MHPSS services and mental health needs

Most survey respondents reported the need to seek healthcare support in the last three months for psychological distress (74.6%) or because of feeling depressed, anxious or stressed (73.9%). Most respondents (95.5%) reported going to the same facility (where they were recruited) as their first choice. In terms of frequency of visits, most respondents visited the facility once per month (45%) or once per week (43%). 85% of respondents considered making appointments for MHPSS counselling and treatment as not or not at all difficult, whereas 13.4% of them considered this process as somehow difficult. When asked about their prescriptions in the last three months, 24.2% of the participants were prescribed medications by their MHPSS service providers. Among them, about 44% reported difficulties to get their medicines.

**Fig. 1:**
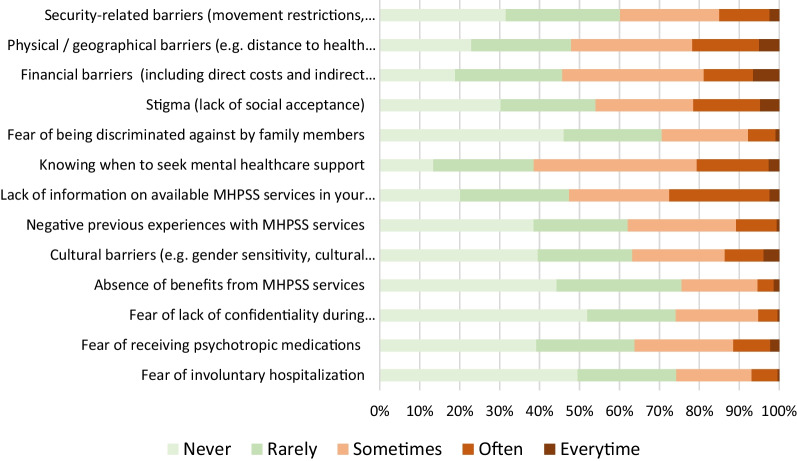
Access barriers to MHPSS services in Northwest Syria (N = 462)

When asked to assess the extent to which they have encountered barriers to MHPSS services in the last three months, respondents reported major issues with knowing when to seek mental health support and the lack of available information about MHPSS services in their communities, followed by physical and financial barriers. For instance, about 61% of respondents reported having difficulties to identify when to seek MHPSS services—either ‘often or every time’ (21%—dark orange in Fig. 1) or ‘sometimes’ (40.7%—light orange in Fig. 1). Moreover, physical barriers and financial barriers were reported by 52% and 54% of respondents respectively, with about 20% of respondents reporting encountering these barriers often or every time. Stigma and cultural barriers were also major barriers to care, with 46% and 37% of respondents reporting to have encountered those barriers at least sometimes. The absence of benefits from MHPSS services and the lack of confidentiality during MHPSS consultations scored as the lowest barriers in our dataset.

In the bivariate analysis assessing the differences in access to services between healthcare workers and other beneficiaries, better access to services was identified by the former. About 95% of health workers considered that making appointments for MHPSS services are not difficult (including 58% being not at all difficult), compared to 74% among other beneficiaries (including 26% of answers being not at all difficult) (*p*-value < 0.001); about 64% of health workers rarely or never encountered physical or financial barriers to care, compared to about 30% of other beneficiaries (p < 0.001); 85% of health workers rarely or never had a perception of ineffectiveness of MHPSS services affecting their access to care, compared to 65% of other beneficiaries (p < 0.001).

#### Mental health outcomes

Almost all survey respondents noted an improvement in their mental health two months after visiting the facility, with about 17% who considered their mental status very improved. In the bivariate analysis, this latter percentage differs between health workers and other beneficiaries (21.5% vs 12.7%; *p* = 0.010). In terms of productivity, ability to work or learn and social interactions, about 85% of respondents reported either good or very good levels. Higher percentages of positive answers were also reported for these two variables among health workers compared to other beneficiaries (see Table 2). Overall, 84% of our survey respondents reported a good or very good mental health status, with statistically significant difference between the study sub-groups (91% among health workers vs 77.1% among other beneficiaries).

**Table 2:** Bivariate analysis table showing the association between the type of beneficiaries and mental health treatment outputs among the sample of MHPSS beneficiaries in NWS (n = 462)

Variables	Healthcare workers (n, %)	Other beneficiaries (n, %)	Total	*P*-value
			**(n, %)**	
Symptoms and concerns (sleeping problems, feeling down/anxious, etc.)				**0.01**
Very improved	50 (21.5)	29 (12.7)	79 (17.1)	
Improved	181 (77.7)	191 (83.4)	372 (80.5)	
Same	2 (0.9)	7 (3.1)	9 (1.9)	
Not improved	0 (0)	2 (0.9)	2 (0.4)	
Productivity, ability to work/learn				** < 0.001**
Very good	59 (25.3)	21 (9.2)	80 (17.3)	
Good	161 (69.1)	154 (67.2)	315 (68.2)	
Fair	13 (5.6)	51 (22.3)	64 (13.9)	
Bad	0 (0)	3 (1.3)	3 (0.6)	
Ability to take care for others and make social interactions				** < 0.001**
Very good	68 (29.2)	29 (12.7)	97 (21.0)	
Good	151 (64.8)	152 (66.4)	303 (65.6)	
Fair	14 (6.0)	43 (18.8)	57 (12.3)	
Bad	0 (0)	4 (1.7)	4 (0.9)	
Very bad	0 (0)	1 (0.4)	1 (0.2)	
Mental health state/"your abilities to cope with the normal stresses of life, work productively and fruitfully, and to make a contribution to your community"				** < 0.001**
Very good	67 (28.8)	40 (17.5)	107 (23.2)	
Good	145 (62.2)	136 (59.6)	281 (60.8)	
Fair	21 (9.0)	44 (19.3)	65 (14.1)	
Bad	0 (0.0)	8 (3.5)	8 (1.7)	
Very bad	0 (0.0)	0 (0.0)	0 (0.0)	

#### Quality of care and patient satisfaction

When asked to assess the quality of MHPSS services, survey respondents had a positive answer (in agreement or strong agreement) regarding the competency and skills of MHPSS providers, the effectiveness of treatment and the person-centredness of services. They also reported high levels of trust in providers. Same positive findings were reported for the satisfaction of beneficiaries with MHPSS services. About 92% of respondents reported being satisfied with the services. However, about 15% of those surveyed were not sure whether they come back for the same service or continue the follow-up with the same MHPSS service provider if needed. Health workers were more likely than other beneficiaries to be unsure about seeking same MHPSS services (18.8% vs. 11.2%, *p* = 0.01).

#### Acceptability of MHPSS and task-shifting

When asked about their main mental healthcare provider, 67.5% reported being treated by a psychosocial worker, followed by 20.3% treated by psychiatrists and 5.4% treated by psychologists. Among those who received MHPSS services from non-specialized providers, about 96% were satisfied or very satisfied with their experiences and the skills of their providers. Among the same group of beneficiaries, about 56% considered that they would have received a better treatment if they were treated by a specialized MHPSS service provider, such as a psychiatrist or a psychologist, and 33% were not sure about it. In the bivariate analysis comparing patients’ experiences depending on the type of provider (specialized vs non-specialized), there was no statistically significant difference in key features of access to services. However, those who were treated by non-specialized providers had higher percentages for improvement of their health status, but lower percentage of willingness to come back to same services (80.9% vs 93.3 for those who considered a specialized provider as their main provider; *p*-value = 0.002).

## Discussion

**Fig. 2:**
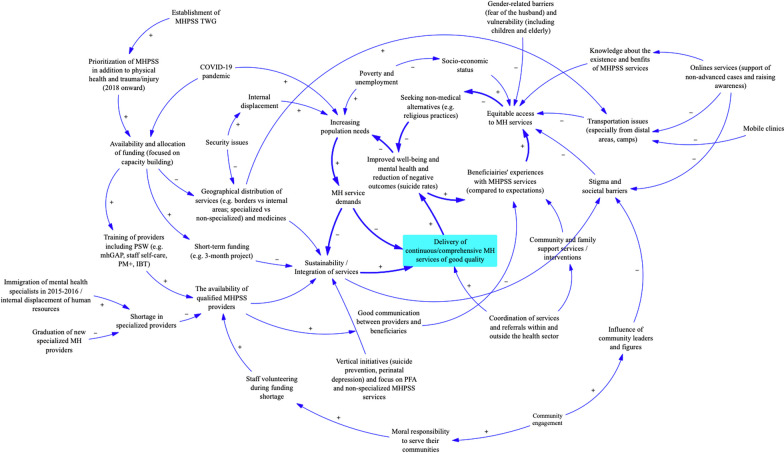
Causal loop diagram showing the dynamics of MHPSS service provision in NWS

Our study explored the provision of MHPSS services in NWS and drew on a variety of data sources to assess the process and outcomes of MHPSS service delivery among the general population and among healthcare workers affected by the conflict. Findings suggested some improvement of MHPSS services in the region over the last few years due to the creation of a specific TWG for MHPSS that contributed to the assessment of MHPSS needs of communities and the support of the MHPSS response. The key elements of this response were: (1) training non-specialized health workers in order to address the shortage in specialized providers; (2) securing funding and coordination of services between different organizations; and (3) addressing gaps in other needed resources such as medicines. While those elements contributed to improving access to services and the quality of services—especially among health workers in need of MHPSS services—findings suggested gaps in the sustainability of services and a need to scale up such interventions in an integrated approach. Targeting those gaps would ensure the delivery of continuous and comprehensive services and improve the mental health status of communities and affected health workers, and therefore reduce pressure on the health system—as shown by the reinforcing loops in the causal loop diagram summarizing the dynamics of MHPSS service provision in NWS (see the arrows in bold in Fig. 2).

Our research indicated that task-shifting was a promising approach to improve the delivery of MHPSS services in NWS. Communities would accept such approaches if deemed effective in reducing their mental health problems. This finding is consistent with the conclusions of a scoping review by Cohen and Yaeger that considered the delivery of MHPSS interventions by lay counsellors in humanitarian settings within low-middle income countries (LMICs) [19]. However, the review highlighted the need for a strong framework for implementation and to engage the providers in the evaluation of the approach. Our findings suggest that practitioners and researchers should investigate how to improve and maintain a continuous support of PSWs and CWs, especially in the case of intermittent funding. Research in other humanitarian settings indicates the importance of adequate culturally adapted training materials and continuous support of adequately selected trainees according to relevant criteria (community relationships, motivation, and experience) [18]. Beyond the direct involvement in service delivery, our study identified community engagement at the strategic level of intervention and service design as a key factor to ensure a higher acceptance of services and a reduction of accessibility barriers by reducing mental health stigma.

Another key issue is the pathway to scaling up mental health interventions in NWS. A systematic review by Troup et al. explored scaling up pathways in LMIC affected by humanitarian crises and noted similar challenges to our study context, i.e. adoption of a horizontal pathway of service replication with long-term sustainability concerns [20]. The review also stressed the importance of greater attention to the vertical dimension of scale up by addressing the health sector-related factors to ensure an integration of services within routine delivery systems. This study also sheds some light on the provision of MHPSS services in NWS from the perspective of health system resilience [21]. Our study suggests that the health sector in NWS showed absorptive capacities to address increasing mental health needs in the context of reduced MHPSS response due to several shocks (including displacement); this was achieved by investing in the capacity of non-specialized human resources and supporting basic interventions (e.g., PFA). However, the adaptive and transformative capacities of the sector in relation to a more comprehensive and sustainable MHPSS provision are yet to be strengthened.

Evidence from this study also suggests that priority actions need to be undertaken for better MHPSS service outputs and outcomes. First, the TWG should advocate for mainstreaming of MHPSS service packages and incorporating MHPSS outputs in the reimbursement of routine services to mitigate the intermittent funding of the MHPSS response. This call for sustainable funding models for MHPSS is critical in shock-prone settings such as NWS, especially given the tendency for shocks of different types (such as the COVID-19 pandemic, the cholera outbreak, and the recent earthquake) to shift international donor support away from MHPSS, despite the increasing MHPSS needs among already severely fragile communities [22]. Sustainable funding also breaks the cycle of resource scarcity, including attrition of trained staff, and helps build a resilient health system.

Moreover, an improved coordination between health and with non-health actors could result in an expansion of the scope of MHPSS response to other levels of the IASC pyramid (such as community support, basic services and security), as well as secure an equitable availability of resources. This system-oriented approach would improve the relationship between health providers and communities as beneficiaries’ experiences with delivered services directly affect their health-seeking behaviour and therefore the ability of the health system to retain them. Health partners need to ensure that the different activities and interventions feed into a system strengthening approach [23], despite the fragility and instability of the context. For instance, there is a need to continuously implement and oversee the task-shifting approach and the delivery of services by low-cadre staff, while adapting long-term approaches to address the gap in specialized services (e.g. engaging psychology students in the MHPSS response and building their practical experience).

Improving the mental health and wellbeing of health providers is another key priority in NWS. Challenges included the limited number of remaining health workers, who have been facing attacks on health facilities, traumatic experiences (such as torture) and stressful events related to the loss of relatives and hard life circumstances for them and their families [5]. These exposures led to increasing mental health problems (such as anxiety) among health workers themselves, who struggled to safely provide healthcare to communities [7]. Our focus on the health of human resources for health in this paper is both a moral and strategic obligation to improve the sustainability and resilience of health services in NWS. Given the specific challenges that health providers face in accessing mental health services (including increased stigma, confidentiality issues, and fear for adverse career impacts and capacity to provide care) [24, 25], the lower level of perceived access barriers and the higher satisfaction rates among healthcare workers accessing services are encouraging, and suggest that this model of MHPSS provision may be well-suited for ensuring access among this key population.

### Strengths and limitations

This mixed methods study followed an inclusive and participative approach, allowing researchers to capture a broad scope of evidence on MHPSS services in NWS and their influencing factors through the involvement of both MHPSS service users and providers in the study. However, we acknowledge that this study might have some selection bias due to the adopted convenience approach. For instance, we might have had a distortion of the population towards the less vulnerable categories of communities (those within the health system already, i.e. those who already accessed the MHPSS services) and towards specific NGO and TWG representatives because the data collection agency was an NGO providing MHPSS services and a member of the MHPSS-TWG. This characteristic of data collection might have also increased the risk of social desirability bias and other information biases (e.g., overestimation of the accessibility to MHPSS services due to the recruitment of participants from MHPSS facilities). However, this risk was mitigated by conducting several interviews and facilitating the GMB workshop by the independent research team itself. Moreover, our data analysis approach (including the bivariate analyses of the survey data and the development of the causal loop diagram) allowed the extraction of inferences which are less likely to be influenced by information biases. As for the impact of the different interventions, its assessment was rather based on the perceptions of participants due to the lack of baseline data for similar indicators (such as satisfaction, mental health outcomes) against which the study findings could be compared.

## Conclusion

This study explored the provision of MHPSS services in NWS, which is a fragile humanitarian setting, through a holistic approach involving different methods and targeting service providers and users. It identified gaps such as intermittent funding and lack of specialized health workforce, but also opportunities that had been taken to improve the MHPSS response, such as through the coordination mechanism and task-shifting approaches. Strikingly, health workers affected by conflict appeared to particularly benefit from the improved MHPSS response. Continuous efforts are needed to scale interventions up and improve the continuum of care in the context, which is still receiving shocks such as cholera outbreaks and chronic stressors. Further research should explore in more detail those key barriers and opportunities to improve the MHPSS response in NWS, specifically in terms of health financing and governance of the response (e.g. to investigate the decision-making processes within and outside the MHPSS-TWG; and to assess funding arrangements of the MHPSS response in NWS).

## Data Availability

Data collected for this study may be provided by the corresponding author upon reasonable request.

## Appendix 1

See Table

**Table 3:** Interview informants and workshop participants’ characteristics

Activity	Category of informants/participants	Number of people	Gender distribution of informants/participants
GMB Workshop	Health managers and field supervisors/coordinators	10 Participants	10 men
	Health care providers (doctors, nurses and PSWs)	5 Participants 2 PSW 1 Midwife 1 Clinical psychologist 1 Psychiatrist	3 men and 2 women
Semi-structured interviews	Representatives of NGOs supporting MHPSS initiatives and members of the MHPSS technical working group	8 informants	6 men and 2 women
	Health professionals working in primary health care centres (PHCCs) and/or mobile mental health units (MMUs) in addition to the non-specialized MHPSS facilities that are connected with PHCs and located within Azaz, Idlib and Afrin. The participants will include doctors, midwives, and other PSWs	12 Informants 2 PHC managers 2 mhGAP doctors 6 PSWs 1 Psychologist 1 Psychiatrist	9 men and 3 women
	Beneficiaries from the host communities (including health workers affected by attacks)	Total: 12 participants	–
	Beneficiaries from the IDPs that have been displaced due to the war conflict	Total: 12 participants	–

3.

## Abbreviations

CLD: Casual loop diagrams
COVID-19: Coronavirus disease 2019
CWs: Community workers
GMB: Group model building
HCWs: Healthcare workers
IASC: Inter-Agency Standing Committee
IDPs: Internally displaced persons
LMICs: Low-middle income countries
M&E: Monitoring and evaluation
mhGAP: Mental Health Gap Action Programme
MHPSS: Mental health and psychosocial support
MHPSS TWG: MHPSS Technical Working Group
MMU: Mobile Medical Unit
NGOs: Non-Governmental Organizations
NWS: Northwest Syria
PFA: Psychological first aid
PHC: Primary healthcare
PM+: Problem Management Plus
PSWs: Psychosocial workers
PTSD: Posttraumatic stress disorders
TWG: Technical Working Group
WHO: World Health Organization
